# Diagnostic Advances and Public Health Challenges for Monkeypox Virus: Clade-Specific Insight and the Urgent Need for Rapid Testing in Africa

**DOI:** 10.3390/diagnostics15232991

**Published:** 2025-11-25

**Authors:** Caroline N. Sambo, Amanda Skepu, Nolwandle P. Nxumalo, Ketlareng L. Polori

**Affiliations:** 1 Human Diagnostic Platform, Chemicals Cluster, Council for Scientific and Industrial Research, Pretoria 0001, South Africa; 2 Department of Life and Consumer Sciences, College of Agriculture and Environmental Sciences, University of South Africa, Private Bag X6, Florida, Johannesburg 1710, South Africa; 3 Food Safety Group, Advanced Agriculture Food Cluster, Council for Scientific and Industrial Research, Pretoria 0001, South Africa; nnxumalo@csir.co.za; 4 Faculty of Health Science, Division of Public Health, University of Free State, Bloemfontein Campus, 205 Nelson Mandela Drive, Park West, Bloemfontein 9301, South Africa; polorikl@ufc.ac.za

**Keywords:** Monkeypox virus, Men sleeping with Men, point-of-care, Clade I, Clade II, infectious disease

## Abstract

**Background:** Monkeypox (MPX), caused by the Monkeypox virus (MPOX) of the Orthopoxvirus genus, has re-emerged as a significant global health threat. Once confined to Central and West Africa, the 2022–2025 multi-country outbreaks, predominantly caused by Clade IIb, demonstrated sustained human-to-human transmission and global spread. **Objective:** This review summarizes current knowledge on MPX virology, epidemiology, clinical presentation, and diagnostic technologies, with a focus on innovations supporting rapid and field-deployable detection in resource-limited settings. **Methods:** The recent literature (2019–2025), including peer-reviewed studies, WHO and Africa CDC reports, and clinical guidelines, was critically reviewed. Data were synthesized to outline key developments in diagnostic methodologies and surveillance approaches. **Results:** MPX comprises two genetic clades: Clade I (Congo Basin) and Clade II (West African), which differ in virulence and transmission. Clade IIb is associated with sexual and close-contact transmission during recent outbreaks. Clinical manifestations have shifted from classic disseminated rash to localized anogenital lesions and atypical or subclinical infections. RT-PCR remains the diagnostic gold standard, while emerging assays such as loop-mediated isothermal amplification (LAMP), recombinase polymerase amplification (RPA), and CRISPR/Cas-based platforms show promise for rapid point-of-care (POC) testing. Complementary serological tools, including ELISA and lateral flow assays, enhance surveillance and immune profiling. **Conclusions:** The resurgence of MPX highlights the urgent need for accessible, sensitive, and specific diagnostic platforms to strengthen surveillance and outbreak control, especially in endemic and resource-constrained regions.

## 1. Introduction

Emerging infectious diseases remain a significant and growing concern, particularly those of zoonotic origin. Among these diseases is Monkeypox (MPX), caused by the MPOX virus, a DNA virus belonging to the Orthopoxvirus, which is part of the Poxviridae virus. The Orthopoxvirus genus includes the eradicated variola virus, which was responsible for causing smallpox and was eliminated by the smallpox vaccine [1,2]. Although MPX is not regarded as high-risk as the smallpox virus, it remains a public health concern due to its endemic presence in various countries, particularly in Africa [2]. It is a re-emerging virus that has attracted attention for its potential to cross borders. The first MPX case was identified in 1958 on monkeys that were kept for research, and the first human contact case was diagnosed in a 9-year-old child in the Central Democratic Republic of the Congo in 1970 [3]. Additionally, further human cases were identified in this country between 1976 and 1977 [4]. Instances of MPX in humans have also been reported in other countries, including Liberia, Nigeria, the Central African Republic, Gabon, the Ivory Coast, Sierra Leone, Cameroon, and Sudan. In 2003, the western United States of America reported its first human case, attributed to a patient’s travel history to endemic areas [5]. Approximately 129,172 laboratory-confirmed MPX cases were reported globally in 2022 alone. This number relates to the multi-country endemic outbreaks that started in May 2022 and involved 130 countries by early 2025, which were associated with Clade IIb [6]. The confirmed MPX cases Clade Ib from 2022 were initially reported from African countries, particularly the Democratic Republic of Congo (DRC), with 8027 cases and 6962 reported cases for 2024 alone [7,8]

The World Health Organization (WHO) declared a Public Health Emergency of International Concern in July 2022 because of a sudden rise in Mpox cases linked to sexual transmission [5,9]. By April 2025, the WHO recorded 32,147 laboratory-confirmed cases and 126 deaths across 27 African member states, with over 39,800 cases reported in Africa since the start of 2025 [10]. Additionally, as of 1 June 2025, the WHO has reported a total of 4798 confirmed cases and 21 deaths globally, with 50 cases reported recently and no new cases reported since the end of June 2025. As it stands, the African continent has the highest number of cases and deaths reported between 1 January 2025 and 1 July 2025, with 28,152 laboratory cases confirmed and 113 deaths [11].

## 2. Virology and Transmission

**Figure 1:**
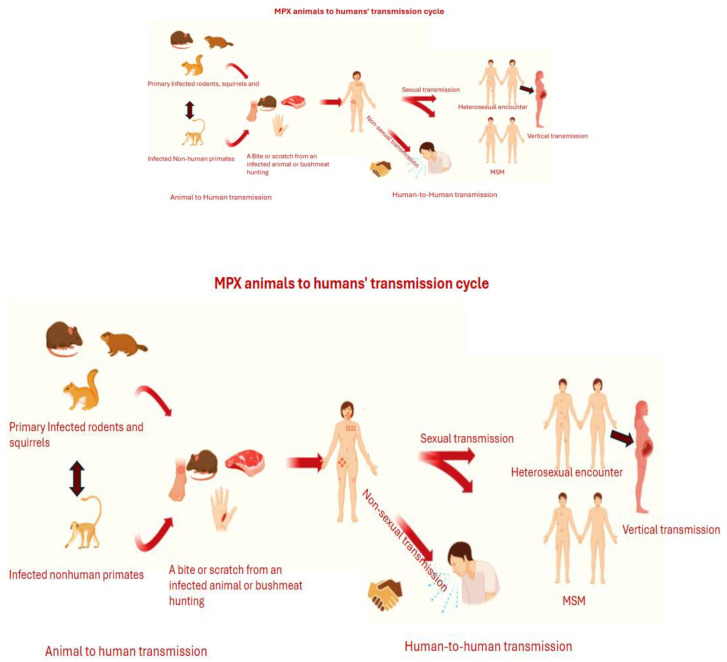
MPX transmission cycle from animals to humans and human-to-human transmission. Created in BioRender. Sambo, C. (2025) https://BioRender.com/o4zx92w (accessed on 29 October 2025).

There are two types of clades for MPX, namely, Clade I with subclades (Ia and Ib), and Clade II with subclades (IIa and IIb). These clades were initially denominated as Central African and West African clades, respectively. They exhibit distinct pathogenic, geographic, and genomic features that influence adaptation, virulence, and transmission [12]. In comparison, Clade I is regarded as more virulent and fatal than Clade II, with a fatality percentage of 10% and 4%, respectively [13]. It has recently been reported that Clade IIb is likely transmitted through human-to-human contact. This mode of transmission includes sexual intercourse involving the exchange of bodily fluids, such as semen, as well as close contact by touching, mouth-to-mouth, mouth-to-skin, through respiratory droplets, and via undisinfected objects like bedsheets, clothing, or sex toys [3,14,15]. Men sleeping with Men (MSM) are regarded as the most at-risk group of contracting the disease. Subsequently, Clade I is zoonotic, whereby its mode of transmission involves humans being in close contact with animals, including nonhuman primates, rodents, tree squirrels, dormice, Gambian pouched rats, and rope squirrels; see Figure 1 [16,17,18,19]. However, ref. [17] note that although Clade I has traditionally been characterized by animal-to-human transmission, recent reports indicate emerging human-to -human transmission particularly associated with sexual activity among MSM mirroring patterns observed in Clade II.

## 3. Epidemiology

Before MPX was regarded as an international endemic, outbreaks were primarily restricted to Central Africa and West African countries, including the DRC, Nigeria, and Cameroon [2]. In this case, the virus was strictly zoonotic, meaning that humans contracted it by coming into close contact with animals. Since these countries are surrounded by rainforests with various species, people have increasingly engaged in hunting, butchering, and consuming animals, which raises the risk of zoonotic transmission and outbreaks [20,21]. Africa is known for its stunning tourist attractions, and encroachment on wild habitats, and this further increases human exposure to animals in reservoirs; consequently, the virus has been introduced to other countries by travellers and tourists. Another contributing factor to human–animal conflict is environmental and ecological shifts, as deforestation, urbanization, agricultural activities, and climate change, including heavy rainfall and flooding, have brought humans closer to wildlife, thereby increasing the risk of zoonotic transmission, as evident in Central Africa [2,20,21]. Demographic and socioeconomic factors, such as population growth and migration, also play a role. This leads to a higher density of people in smaller environments, resulting in more frequent human interactions and, consequently, the spread of the virus. Low-income communities, which often rely on bushmeat for consumption and practice minimal hygiene after handling wild animals, are also contributing to the issue.

Reports from the African Centres for Disease Control and Prevention (Africa CDC) indicate a trend of increasing MPX cases in African countries. From 2022 to August 2024, 42,874 confirmed MPX cases and 1512 (3.5%) deaths have been reported in 15 Union Member States (AUM): Benin, Burundi, Cameroon, Central African Republic, DRC, Congo, Egypt, Ghana, Liberia, Morocco, Mozambique, Nigeria, Rwanda, Sudan, and South Africa [22,23]. In 2023 alone, 14,957 cases and 39 deaths (4.9%) were reported in 7 AUMS, marking a 78% increase from 2022 [22], and 2024 also showed an increase in the number of reported cases in 13 AUMS, totalling 18,737 with 541 deaths, representing an increase of 104% in cases and 10% in fatalities [23]. These MPX cases were observed to peak during mid-year, between June and August, in the winter season, when low temperatures are experienced, along with human mobility and agricultural cycles in certain seasons. This suggests that the increasing trend may be due to changes in temperature, humidity, and rainfall during seasonal shifts. Researchers have also reported that cooler and more humid conditions may allow the virus to survive longer in the environment, thereby increasing the likelihood of transmission.

Africa faces significant challenges due to inadequate surveillance and minimal health infrastructure. In many instances, scientists depend on paper-based manual data collection, leading to inconsistencies or incomplete data, as disease surveillance is often underfunded and fragmented; however, despite all the limitations and challenges that the countries had encountered, researchers were able to detect the virus, identify the clades, and characterize the endemic [24,25]. Furthermore, routine health data is seldom analyzed, which diminishes the timeliness and accuracy of outbreak detection. Although the characteristics of MPX have been identified, surveillance of animal reservoirs remains minimal [24]. Reports indicate that African countries suffer from a shortage of healthcare workers, which contributes to limited data and specimen collection, patient management, and case identification. Additionally, some patients are misdiagnosed, underrecognized, and experience delays in reporting [25]. Another contributing factor is the restricted access to MPX therapeutics, as supplies are prioritized for high-income countries, leaving rural African areas often unserved or underserved [26].

Therapeutics such as Tecovirimat, generally used to treat smallpox, Cidoforvir, and Brincidofovir, which were originally developed for other viral infections, have emerged as important for the management of severe MPX cases, particularly in immunocompromised patients [27,28,29]. Although there is a selection of drugs that might be used for MPX management, no drug has been formally licenced for MPX specifically yet [27]. The availability of therapeutics has primarily contributed to the reduction in severe disease outcomes, complications, and hospitalization rates, impacting the overall severity of the endemic [29,30,31].

The use of smallpox vaccination, particularly the JYNNEOS vaccine, has assisted in the reduction in susceptibility in high-risk populations, as it provides cross-protection against MPX [27,29,32,33]. Countries that historically had smallpox vaccinations demonstrate lower MPX case rates and mortality rates regarding the recent outbreak [29,33]. Despite the low full vaccination coverage, the outbreak was controllable; this may be due to vaccination combinations, behavioural changes, natural immunity, or asymptomatic infections [29,33].

Contact tracing and isolation of infected individuals helped limit secondary transmission, particularly in non-endemic countries, where the transmission route was primarily linked to close physical contact, which includes sexual activities [31,33,34]. The implementation of risk communication, reaching out to the high-risk population, especially MSM, and advice on the use of condoms also reduced the spread [31,35]. Contact tracking was significant in reducing transmission and identifying asymptomatic or pre-symptomatic cases, which further prevents wide community spread [29,33].

## 4. Clinical Presentation

**Figure 2:**
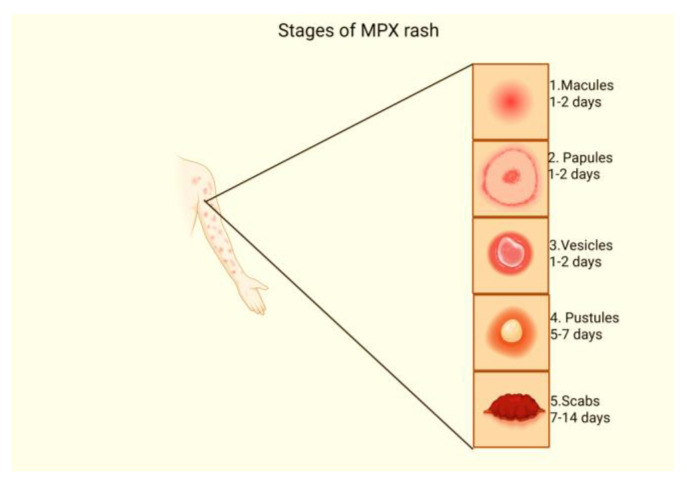
MPX’s noticeable clinical presentation is the presence of lesions that develop in different stages from macules to scabs, which also differ in their duration before resolving. Created in BioRender. Sambo, C. (2025) https://BioRender.com/r0l8hs4 (accessed on 29 October 2025).

Symptoms typically appear within a timeframe of 1 to 20 days after exposure to the virus and generally disappear within 2 to 4 weeks, although they can persist longer in immunocompromised individuals, including older people, children, and pregnant women [15]. In some instances, the virus may be contagious anywhere from 1 to 4 days before symptoms appear and may not be contagious only after all the symptoms have worn off, scars have healed, and new skin has formed. MPX involves four stages: incubation, prodrome, rash, and lesion. Firstly, in the incubation stage, a person may feel well with no symptoms and not be contagious, though monitoring is recommended; this stage may last for 5 to 21 days [36,37]. Secondly, in the prodromal stage, one experiences fever, headache, muscle aches, back pain, chills, exhaustion, oral lesions, sore throat, and swollen lymphadenopathy, which is essentially used to distinguish MPX from other poxviruses [38,39,40]. Thirdly, the rash stage begins on the face and spreads to other parts of the body, including the palms and soles, but the recent outbreak often shows initial lesions on anogenital/genital areas. Fourthly, in the lesion stage, lesions are deep-seated, firm, and circumscribed with central depression. It progresses through several stages that include macules, which are discoloured flat spots lasting for 1–2 days, followed by papules, which are elevated bumps that also last for 1–2 days. Vesicles last for 1–2 days and are associated with fluid-filled blisters, followed by pustules, which are pus-filled lesions that last for 5–7 days before scabbing and healing, which resolves in 7 to 14 days; Figure 2 [17,36,41,42,43,44]. In contrast to the common clinical manifestations of MPX, recent reports have shown both typical and evolving symptoms. The difference from 2022 to 2025 shows a broader recognition of atypical presentations and complications. These presentations include a limited number of lesions localized on the perianal areas and genitals, rather than being widespread, exclusive of the classic centrifugal rush pattern that was seen in 2022 [35]. These lesions display a nonsimultaneous development with different lesions at different stages, harmonizing in the same area, such as the pubic area or penis, with minimal or absence of prodromal symptoms in comparison with the first reported clinical presentation [45,46]. The genital lesions can be painless and asymptomatic, with severe edema potentially leading to complications such as urethral strictures; this has been reported with genital involvement [47]. Moreover, co-infections with HIV have been associated with more severe symptomology, including genital ulcers and a more pronounced rash [28,35].

Typical complications arising from MPX include neurological complications such as seizures, confusion, and encephalitis, which have recently been observed in other cases, alongside even more severe complications, including corneal infections leading to vision loss, bacterial skin infections, pneumonia, sepsis, and myocarditis [28,29,48,49]. Proctitis (painful swelling of the rectum), painful swallowing, and difficulties when urinating were also noticeable symptoms in some patients [29]. The disease can be fatal, especially in immunocompromised patients, children, pregnant women, and individuals with poorly controlled HIV [28,29,35]. Formerly from 1970 to 1989, MPX primary affected children under the age of 4 years in the endemic areas; then, the medium age changed to 10 and 12 years between 2000 and 2009, especially with Clade Ib infections, and 18–50-year-old-adults’ cases reported in 2022 to 2025 in sexually active individuals with an increase from 11% (95% CrI: 7–17%) in September 2024 to 51% (95% CrI: 46–55%) in December 2024, before dropping by 6% (95% CrI: 0.3–19%) in April to 25 [34]. However, recent reports tend to show an increasing proportion of children between the ages of 0–4 and 5–9 years old, with a relative risk of 3.45 (95% CrI: 2.56–4.89) and 2.27 (95% CrI: 1.76–4.10), respectively, indicating the spread of MPX to a broader age group [28,34,50]. Furthermore, children tend to be at a high risk of severe disease and complications, as this clade has high mortality [34,51]. MPX virus has so far shown symptoms that are similar to other infectious diseases; furthermore, accurate and early diagnosis is essential for both in optimising individual patient care and ensuring effective public health management.

Early, reliable, and accurate diagnosis enables supportive care, timely treatment, and prevents future complications, especially for high-risk individuals such as children, pregnant women, and those with compromised immune systems. Additionally, misdiagnosis is possible as the recent outbreak demonstrated an initial concentration of lesions in the genital and perianal regions, likely reflecting physical and sexual contact transmission dynamics [15,40,41]. Accurate laboratory confirmations from the aforementioned symptoms are highly important in the identification of viral infection, patient isolation, and contact tracking, which reduce transmission and control the outbreak. The continued rise of MPX cases in African countries signifies the insufficiency of effective viral containment strategies, which have facilitated unchecked transmission within the affected population.

## 5. Diagnostic Testing Approaches

The development of accurate and rapid medical tools is essential for early diagnosis and determining a way forward for human diseases such as MPX. Diagnostics measures for MPX have not yet been established; however, scientists believe that certain diagnostic methods can be utilized to identify the MPX virus in an individual exhibiting the aforementioned symptoms, especially if the person has a history of travelling to endemic areas or has had contact with an infected individual. According to [52], the current outbreak differs from the originally discovered virus in the West African region due to its typical transmission route involving sexual contact; in contrast, the first reported MPX case was mostly zoonotic with no reported cases of sexual contact transmission. [52] further emphasized the importance of health workers considering an alternative diagnostic measure if a patient presents with sexually transmitted infectious diseases associated with a rash, as well as a genital ulcer disease evaluation, to prevent misdiagnosis.

The recommended specimens for laboratory confirmation of MPX are lesion materials that are obtained by swabs on the vesicles or crust from the skin or the mucosal lesions; subsequently, throat swabs can be collected from individuals without visible lesions [13]. Table 1 summarizes the processes and possible laboratory confirmation methods that can be used to diagnose MPX.

### 5.1. Sample Collection and Storage

**Figure 3:**
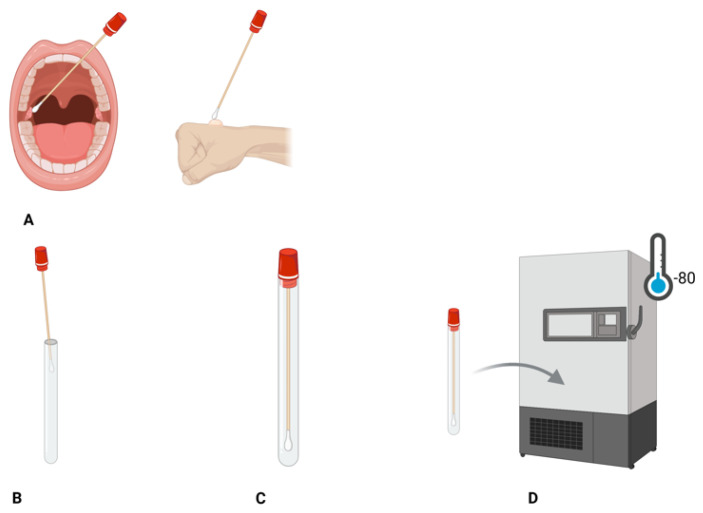
An illustration of the process for sampling and storage of the MPX swabs. (**A**) Swabbing for MPX from at least two lesions from different parts of the body. (**B**) Swab storage in a sterile, dry container. (**C**) Proper labelling of the container. (**D**) Specimen long-term storage in a −80 °C freezer. Created in BioRender. Sambo, C. (2025) https://BioRender.com/3lz2ht0 (accessed on 29 October 2025).

The correct process for specimen collection, handling, and storage is important for the accurate and correct diagnosis of an infection. The recommended sample types for laboratory testing for MPX infection by the WHO include mucosal lesions, preferably vesicular fluid, lesion swabs, genital swabs, and dry swabs; for individuals where lesions are not visible, throat swabs are recommended. However, the throat sample, compared to the other sampling parts, is known to contain a low viral load; therefore, when interpreting the results, that should be taken into consideration (see Figure 3 below) [53]. A recent study published in Infectious Disease Reports supports the use of lesion and rectal swabs for mpox diagnosis, as these sample types show lower median cycle threshold (Ct) values (Ct = 23 and Ct = 22, respectively), indicating higher viral loads compared with throat swabs and urine samples (Ct = 31 and Ct = 30, respectively). Consequently, lesion and rectal swabs are preferable for MPX detection in laboratory settings due to their higher sensitivity and viral load [53,65]. Ethylenediaminetetraacetic acid (EDTA)-treated whole blood may be of assistance in diagnosing MPX, but since viremia occurs in the early stage before lesion formation, this sample may contain a low viral load [38]. MPX is contagious; therefore, a relevant protocol for safety, i.e., PPE and containment of the virus to prevent infection/spreading, must be implemented and adhered to by medical and health professionals at all times [38,53]. Samples must be kept refrigerated at 2–8 °C or frozen at −20 °C and be tested within 7 days [53]. During transportation, samples must be placed in a sterile, tightly sealed primary container, followed by triple packaging, in compliance with the International Air Transport Association guidelines for biological substances category B(UN3373) [66]. The triple-packaging system consists of the primary container, secondary packaging, and an outer shipping container that is designed to resist physical pressure and withstand different environmental conditions [66,67]. For future research and development work, the recommended long-term storage of the samples is in a −80 °C freezer [53]. Ethylenediaminetetraacetic acid (EDTA)-treated whole blood may be of assistance in diagnosing MPX, but since viremia occurs in the early stage before lesion formation, this sample may contain a low viral load [38]. MPX is contagious; therefore, a relevant protocol for safety, i.e., PPE and containment of the virus to prevent infection/spreading, must be implemented and adhered to by medical and health professionals at all times [38,53]. Samples must be kept refrigerated at 2–8 °C or frozen at −20 °C and be tested within 7 days [53]. During transportation, samples must be placed in a sterile, tightly sealed primary container, followed by triple packaging, in compliance with the International Air Transport Association guidelines for biological substances category B(UN3373) [66]. The triple-packaging system consists of the primary container, secondary packaging, and an outer shipping container that is designed to resist physical pressure and withstand different environmental conditions [68,69]. For future research and development work, the recommended long-term storage of the samples is in a −80 °C freezer [53].

### 5.2. Real-Time Polymerase Chain Reaction (RT-PCR)

Nucleic acid amplification testing from skin exudate, genital or vesicle lesions, and crust using RT-PCR is employed in African countries to diagnose MPX due to its high sensitivity and accuracy, which reaches 100% [52,68]. The method does not just focus on MPX but on all the Orthopoxviruses suspected of association [69]. Additionally, the method can distinguish between the MPX clades [70,71], which is essential for tracking and managing outbreaks. This distinction is achieved by targeting unique regions of the genome sequence, utilizing the specifically designed primers for that region [71]. The United States Food and Drug Administration has granted an emergency use authorization to this method [72].

### 5.3. Molecular Point-of-Care Testing (mPOC)

Molecular point-of-care testing for MPX aims to provide early, rapid, and accurate detection of the virus near the patient without the need for laboratory infrastructure and specialized personnel [68,72]. Such testing is critical and useful in resource-limited and remote settings in African countries. The United States Food and Drug Administration (FDA) has granted emergency use authorization for two mPOC tests, but only one is still being manufactured [53]. The method primarily relies on Nucleic Acid Amplification Tests (NAATs) such as isothermal amplification methods, including Loop-Mediated Isothermal Amplification (LAMP), Recombinase-based Isothermal Amplification assay, and PCR platforms [53]. The viral DNA load of MPX (Clade IIb only) and non-variola orthopoxvirus was obtained directly from the human lesion swab specimen [53], and the results were validated by the USFDA-cleared real-time PCR reference standard. Further clinical validations were conducted on samples from patients in the United States who had confirmed positive MPX Clade IIb, corresponding to the laboratory-based PCR reference standard [53,68].

#### 5.3.1. Serological Testing Method

The serological assay method (antigen–antibody) plays a complementary role in MPX diagnosis by detecting antibodies that are generated in response to MPX virus infection from the patient’s serum or plasma [55]. Common primary serological methods include Enzyme-Linked Immunosorbent Assay (ELISA), lateral flow assays (LTFs), immunofluorescence assays (IFA), plaque reduction neutralization testing (PRNT), and others. These methods specifically measure certain immune responses, primarily immunoglobulin M (IgM) and immunoglobulin G (IgG) antibodies, providing information about past and recent infections, thereby aiding in seroprevalence studies [38,44,73]. Age has a significant role in the antibody response of MPX virus, as the old generation born before 1979, who received the historical smallpox vaccine, demonstrates a higher and more stable level of cross-reactive antibody against the MPX antigen and neutralization antibodies, whereas the new generation born after 1979 without the vaccine, shows lower baseline MPX reactive antibodies and only develops mutable antibodies after the infection. Moreover, for this reason, a serological assay diagnostic cut-off may be necessary to avoid false positives in older individuals who may be carrying cross-reactive antibodies from prior smallpox vaccination [74,75]. Although PCR remains the gold standard for MPX diagnosis as it detects the virus directly, the serological test does not detect the virus itself but focuses on the host’s immunological response, making the method more suitable for retrospective confirmations [38,73].

##### Enzyme-Linked Immunosorbent Assay

ELISA is a well-established laboratory-based method that provides a quantitative and qualitative measurement of the antibody level and optimized peptides to ensure high specificity (>90%) and sensitivity (70–89%) [38,55,56,57,73,76]. ELISA testing methods are suitable for large-scale epidemiology in high-risk populations as it is simple to use, convenient, and safe [56]. The specific antibodies IgM and IgG are usually detected within 5–8 days after the appearance of the rash; furthermore, these antibodies can be detected in the serum, whole blood, and fingertip blood samples [56]. Although antibodies obtained from plasma or serum are fairly considered for MPX diagnosis, ELISA tests may come in handy if PCR results are determined to be inconclusive by testing the IgM in a recently infected patient and testing the duplicate IgG in the serum from the first week of infection [55,56,57,76]. For infection confirmation, specimens from both the acute stage and the recovery stage are required to compare the antibody level of IgG [56,77]. Although ELISA has been determined to be safe and easy, it is not recommended for early detection diagnosis as antibodies may take time to appear in the serum [77,78]. Moreover, vaccination interference may result in false positive results from IgG antibodies induced by past smallpox vaccination, complicating the differentiation between neutral MPX virus and past immunization, especially in immunocompromised individuals [68].

##### Lateral Flow Assay

In contrast, LTFAs provide rapid, point-of-care testing capabilities by detecting the viral or host’s antibodies directly from clinical specimens such as saliva or lesion swabs (IgM and IgG) through immunochromatographic detection on a nitrocellulose membrane and providing semi-qualitative or quantitative results in 10–15 min [56]. LFAs are emerging as a promising diagnostic tool for MPX due to their potential for use outside of highly complex laboratories [58]. LFAs’ technology has recently advanced to enhance the sensitivity and user accessibility in field settings using nanoparticle conjugates, and the use of the double antibody sandwich technique on a strip demonstrated no cross-reactivity with other infectious diseases [38,56,58,59,73]. Furthermore, the recently introduced four specific monoclonal antibodies aim to target the A27 MPX protein, which shows a high specificity level of detecting a low viral load of 3 × 10^5^ pfu/mL [58,79]. LFA is simple and portable, with minimal training needed for users, quick to interpret results, and due to its low cost, it enhances its accessibility, especially in low- and middle-income settings. WHO has not recommended the use of rapid tests yet [44], due to limitations in assessing the viral load, low sensitivity, and specificity, which may be due to cross-reactivity with other orthopoxviruses, resulting in false positive results and the influence of the sample buffer compositions used on assay performance, as well as low development of antibodies during the acute phase [55]. Despite its limitations, LFA holds potential for the rapid, point of care testing needed to control the MPX outbreak and can be used as a complementary diagnostic technique with other methods. Research scientists have found a way to use LFA as a readout method with other methods such as Loop-Mediated Isothermal Amplification (LAMP).

#### 5.3.2. Loop-Mediated Isothermal Amplification (LAMP)

Unlike the PCR method, the isothermal amplification method does not require expensive instruments or thermocyclers; it requires a constant temperature, which is ideal for low-income countries, such as Africa, and it is faster and more sensitive. This amplification method utilizes four to six primers with a DNA polymerase that binds specifically to the target complementary sequence genome and amplifies the DNA strand at a constant temperature rate, typically 60–65 °C [80]. The results can be detected/observed through visual colour change, pH indicator, turbidity measurements, fluorescence, or a lateral flow assay [60,80]. The advantages of this method are its simplicity and its ability to provide results within 30 to 60 min, as well as its straightforward workflow, showing its continuity in low- and middle-income countries [38,60]. The method displays a very low detection limit as compared to the qualitative PCR (qPCR) method, often below 20 copies, as well as a rare cross-reactivity with pathogens other than MPX [60,80,81]. LAMP’s minimal equipment and technical requirements make it a viable method in low- and middle-income settings, addressing a critical gap in the diagnosis of MPX.

#### 5.3.3. Loop-Mediated Isothermal Amplification and Lateral Flow Assay (LAMP-LFA)

The LAMP-LFA technique is a diagnostic method that combines rapid visual detection with nucleic acid amplification to meet the needs of POC diagnosis, especially in low- to middle-resource settings with limited laboratory infrastructure. According to [60], the technique specifically targets the MPX virus ATI gene using multiple primers to amplify the viral DNA at a constant temperature for about 40 min. This gene encodes viral inclusion proteins vital for the virus’s structure, ensuring high specificity for the MPX virus only. The amplified DNA mixture is then applied to a lateral flow strip containing capture antibodies that bind to the LAMP amplicons, resulting in visible results that can be read by the naked eye within 3–5 min, indicating the presence or absence of the virus. The advantages of this method include instrument-free interpretation, low operational costs, simplified workflow, and high sensitivity and specificity comparable to PCR, without the need for expensive thermocyclers or fluorescence detection, with a turnaround time of 60 min, making it suitable for field settings. Combining LAMP with LFA-based readout provides a POС-compatible option for low- to middle-resource environments and helps address the gap in rapid diagnostic testing during the current pandemic. For MPX-LAMP-LFA to compensate for its limitations, careful sample preparation and proper storage of reagents are crucial for stability and reproductivity of results [82,83]. Overall, MPX-LAMP-LFA acts as a bridge between traditional diagnostic tools, making it a valuable innovation for MPX control and health readiness.

#### 5.3.4. Recombinase Polymerase Amplification (RPA)

RPA harnesses the recombinase enzymes to facilitate the binding of primers to the homogeneous sequence in the target double-stranded DNA, followed by the displacement of DNA strands, and synthesis at a constant temperature between 37 °C and 45 °C [38]. It has demonstrated a low limit of detection of 16 DNA molecules per microlitre and shows a clinical performance of 95% as well as a sensitivity rate of approximately 100% on human and primate samples tested during the current outbreak [84]. Results can be visualised by fluorescence, lateral flow assays, or gel electrophoresis [38]. Due to the low resource requirements, the method can be deployed in mobile, solar-powered suitcase laboratories for field work.

Further advancements have been discovered for RPA integrated with CRISPR-Cas12a detection for increased sensitivity and specificity. This method shows a low detection rate of one MPX DNA copy per microlitre within 40 min [85]. The integration employs lyophilized reagents for stability and incorporates heating lysis for rapid sample processing in the absence of nucleic acid extraction, facilitating its use in resource-limited settings [86]. A one-pot sucrose-aided RPA-CRISPR assay has also been reported, demonstrating high specificity and sensitivity for MPX infection. This method combines isothermal amplification and CRISPR-mediated fluorescence detection and allows visual readouts of results, suitable for convenient and rapid testing [87].

### 5.4. CRISPR/CAS-Based Technology

This recently developed method provides rapid and sensitive MPX results comparable to those obtained with qPCR. This easy-to-use medical device offers highly accurate readings and is suitable for settings without complex equipment [54,72]. Although still under research, the CRISPR/CAS-based technology is thought to be an alternative approach to nucleic acid detection techniques due to its simple device structure, high specificity and sensitivity, and compatibility with other techniques’ readouts, such as LFA or fluorescence [72]. CRISPR/CAS-based platforms such as SCOPE (cas13a), MPXV-RCC (cas12a), and Kairo-CONAN (cas3) show the potential to revolutionize MPX diagnostics, especially in low-income or limited-resource settings [88,89,90,91,92,93]. While these platforms are promising, they still require validation, regulatory approval, and integration into health systems to control and contain the recurring MPX outbreak [85,89,92,94].

### 5.5. Viral Culture

Viral culture entails isolating the MPX virus from clinical specimens by infecting other cell lines and observing the resulting cytopathic effects. Suitable specimens include lesion materials such as variola fluids, crusts, and biopsies, while urine, swabs, and semen may be utilized for research purposes [56,72]. This method requires high-containment laboratories, Biosafety Level 3 (BSL-3) or higher, due to the virus’s highly infectious nature, which poses risks to laboratory personnel. Consequently, only labs with the requisite experience and containment facilities are authorized to conduct viral culture [53,67,68]. Results can be read out and observed using a Transmission Electron Microscope (TEM) and Light Microscopy, observing the cytopathic effect (CPE) [61,95]. Given its complexity, biosafety risks, and turnaround time requirements, this approach may not be suitable as a routine diagnostic tool, but it can be employed in laboratory research, antiviral susceptibility testing, and test confirmation [38,96]. It can also play an important role in studying the host–cell interaction and viral replication kinetics, as well as diagnosing poxviruses, including MPX, if the nucleic acid tests give false negatives or fail [61].

### 5.6. Whole Genome Sequencing

Whole genome sequencing is a diagnostic tool that targets an individual’s entire genetic code and offers a comprehensive approach to identifying the causes of health problems and characterizing them, as well as surveillance and monitoring of viral evolution and variants. This method is considered powerful due to its ability to detect unknown transmission chains, the genetic variants and strains, and the origin of an outbreak [72]. Whole-genome sequencing uses sequencing protocols such as metagenomic next-generation sequencing, which enables unbiased detection of the genetic materials found in the clinical sample and monitors emerging variants [28,97]. Amplicon-based and multiplexed methods enable high-quality sequencing of the MPX genome from the clinical materials to the MPX virus with high sensitivity and coverage, including samples with low viral load, such as throat swabs [98]. The turnaround time for results ranges from 12 to 48 h, depending on the resources and sequencing method [64,98]. The need for high high-cost reagents, extensive computational resources, and significant infrastructure restricts its viability as a large-scale POC diagnostic tool for MPX [72].

## 6. Diagnostics Limitations

### 6.1. Limited Testing Coverage

In 2024, only 37% of suspected cases in low-income African nations like the DRC were tested, falling short of the 80% threshold recommended for controlling the current outbreak [99,100]. Many regions, particularly rural areas, face significant barriers to testing access, resulting in many individuals remaining untreated. Since the first human case of MPX in the DRC 54 years ago, there are still substantial gaps in understanding transmission routes, the virus’s natural history, and the risk of MPX across Africa [26].

### 6.2. Gaps in POC and Rapid Diagnostics

Although several POC and rapid diagnostic tools are available for MPX, they are still under development and currently do not meet the WHO’s sensitivity requirements for effective deployment in Africa [101], which limits the ability to contain the outbreak in the region.

### 6.3. Reliance on Imported Kits and Medications

Most diagnostic kits come from higher-income, international countries, leading to procurement delays, supply chain issues, and elevated costs [102]. This complicates the acquisition of the instruments and laboratory chemicals necessary for diagnosing MPX. Important medications, including pain relievers, antimicrobials, treatments for skin lesions, antipyretics, and intravenous therapies, are crucial for managing symptoms, minimizing complications, and reducing mortality rates among those who test positive [103,104,105]. Since these drugs are imported, they take time to arrive in Africa, further delaying efforts to contain the virus.

### 6.4. Lack of Clade Multiplexing

Many diagnostic tools on the market struggle to distinguish between clades, often only identifying one at a time. Currently, these tools can detect MPX Clade IIb but fail to recognize Clade Ib, which is prevalent in the DRC and associated with higher mortality rates than Clade IIb. If this situation persists, it could result in ongoing reported cases and an increase in fatalities.

### 6.5. Limited Laboratory Facilities and Discontinuation of the Smallpox Vaccine

African nations face significant healthcare challenges, with healthcare professionals possessing limited knowledge about the MPX virus, as well as restricted access to detection technologies, treatment options, and diagnostic skills compared to other regions [106]. According to the CDC, the smallpox vaccine can reduce MPX symptoms within two weeks of exposure, though it does not prevent the disease [69,106]. Despite this, the vaccine is either unavailable to the public or not administered to patients. The first-generation smallpox vaccine (VACV) is not recommended, as it fails to meet current safety and manufacturing standards [106]. Reports of safety for the second-generation vaccine (ACAM2000) are lacking safety data for the second-generation vaccine (ACAM2000 are limited, particularly among individuals with HIV, and its use is associated with serious concerns related to events like myopericarditis [69,106]. The third-generation JYNNEOS vaccine has received manufacturing authorization from the US FDA and is approved for individuals aged 18 and older [17,69,106]. However, this vaccine is currently limited to countries such as Canada, the United States, and Ecuador, and will take time to become accessible in African nations [17].

## 7. Conclusions and Future Recommendations

Current diagnostic tools for MPX mostly rely on molecular diagnostic techniques, such as PCR, which is regarded as the gold standard globally due to its high specificity and sensitivity, and other methods, such as sequencing, for its ability to differentiate the clades. There are advances in laboratory-based diagnostics that provide confirmatory tests as well as epidemiological surveillance; however, there is still a gap in the development and availability of Rapid Diagnostic Tests (RDTs) that can demonstrate quick POC results.

The absence of validated RDTs delays diagnosis in clinical and field settings, especially in low- and middle-income countries such as African countries. This results in restrictions to early case identification, the isolation of infected patients (thus increasing transmission cases), the initiation of treatment, and public health intervention, thus affecting outbreak containment. Currently, the only regulatory approvals focus on PCR and antigen–antibody rapid tests, which have not yet met the required clinical sensitivity and specificity recommended standards. Furthermore, there is an urgent requirement for the development and validation of easy-to-use, accurate, rapid diagnostic assays for MPX. There are already existing molecular methods and emerging technologies that may complement these RDT tools, which may enable faster decision-making directly at the POC and improve disease management, especially in low- and middle-income countries.

## Figures and Tables

**Table 1:** Diagnostic detection methods for MPX.

Diagnosis Approaches	Sample Required	Setting	Time	Feature
PCR	Skin lesion swabs, Oropharyngeal (throat) swabs	Laboratory setting/POC	45–60 min	Gold standard [53]
LAMP	Skin lesion swabs	POC	30–60 min	Isothermal, high sensitivity
RPA	Skin lesion swabs	POC	3–15 min	Isothermal, high sensitivity, comparability with multiplex [38]
CRISPR/CAS	Skin lesion swabs	POC	45 min	Atomic sensitivity level, integrated with POC platforms, single-based specificity for the target genome [54]
ELISA	Serum, plasma	POC	2–4 h	Processing large number of samples at once, high sensitivity and specificity [55,56,57]
LFA	Serum, plasma, saliva	POC	10–15 min	RDT, fast, user-friendly and simple, minimal equipment requirement, portable [58,59]
MPX-LAMP-LFA	Lesion swabs, blood	POC	60 min	No cross-reactivity, high specificity, simple and portable [60]
Viral isolation	Skin lesion swabs	Laboratory setting	2–6 days	Viral identification, confirmatory [61]
Whole genome sequencing	Skin lesion swabs	Laboratory setting/POC	12–48 h	Reducing cross-reactivity, high quality, and accuracy [62,63,64]

Loop-mediated Isothermal Amplification (LAMP); Point of Care (POC); Recombinase Polymerase Amplification (PCR); Clustered Regularly Interspaced Palindromic Repeats (CRISPS/CAS); Enzyme-Linked Immunosorbent Assay (ELISA); Lateral Flow Assay (LFA); Mpox-Loop-Mediated Isothermal Amplification-Lateral Flow Assay (MPX-LAMP-LFA).

## Data Availability

No new data were created or analyzed in this study. Data sharing is not applicable to this article.
